# DNA-Platinum Thin Films for Use in Chemoradiation Therapy Studies

**DOI:** 10.1155/2012/923914

**Published:** 2011-10-02

**Authors:** Mohammad Rezaee, Elahe Alizadeh, Darel Hunting, Léon Sanche

**Affiliations:** Groupe en Sciences des Radiations, Départment de Médecine Nucléaire et Radiobiologie, Faculté de Médecine et des Sciences de la Santé, Université de Sherbrooke, Sherbrooke, QC, Canada J1H5N4

## Abstract

Dry films of platinum chemotherapeutic drugs covalently bound to plasmid DNA (Pt-DNA) represent a useful experimental model to investigate direct effects of radiation on DNA in close proximity to platinum chemotherapeutic agents, a situation of considerable relevance to understand the mechanisms underlying concomitant chemoradiation therapy. In the present paper we determine the optimum conditions for preparation of Pt-DNA films for use in irradiation experiments. Incubation conditions for DNA platination reactions have a substantial effect on the structure of Pt-DNA in the films. The quantity of Pt bound to DNA as a function of incubation time and temperature is measured by inductively coupled plasma mass spectroscopy. Our experiments indicate that chemical instability and damage to DNA in Pt-DNA samples increase when DNA platination occurs at 37^*°*^C for 24 hours, the condition which has been extensively used for in vitro studies. Platination of DNA for the formation of Pt-DNA films is optimal at room temperature for reaction times less than 2 hours. By increasing the concentration of Pt compounds relative to DNA and thus accelerating the rate of their mutual binding, it is possible to prepare Pt-DNA samples containing known concentrations of Pt while reducing DNA degradation caused by more lengthy procedures.

## 1. Introduction

Clinical studies have shown that concomitant treatment with chemotherapeutic drugs and radiotherapy often leads to a higher rate of survival and local tumor control compared to nonsynchronous treatments [1, 2]. Platinum chemotherapeutic drugs are commonly used in concurrent chemoradiation therapy (CRT) for treatment of solid tumors [3]. Although it is clear that platinum drugs and radiation in CRT modalities increase tumor cell killing, improve locoregional control of tumors, and enhance patient survival [4, 5], the optimum schedule of the combination and the underlying mechanisms of their synergistic action have not been yet defined [6, 7]. Since DNA is the common target of both radiation and platinum chemotherapeutic agents, most studies have focused on the structural and functional alteration of DNA resulting from the combination [8, 9]. One possible mechanism responsible for the observed synergy is enhancement in immediate (secondary) species induced by primary radiation in the vicinity of the binding site of the platinum compounds (Pt compounds) to DNA [10, 11]. The most abundant of these secondary species are electrons with the most probable energy of 9-10 eV [12]. Studies on the interaction of secondary low energy electrons (LEEs) with DNA have elucidated some of the fundamental mechanisms leading to DNA damage [13]. However, owing to the short range (~10 nm) of LEE in biological matters, such studies must be performed on very thin DNA films of similar thickness. Pt-DNA thin films could provide an experimental approach to investigate the direct effects of the secondary electrons and other short-range particles (or secondary species) on DNA in the presence of Pt compounds. Such investigations could disclose mechanisms underlying the synergistic effect between the radiation and the drug, which may have implications for the optimization of protocols in CRT as well as in the design and development of new chemotherapeutic and radiosensitizing drugs [14].

Dry thin films of bacterial plasmid DNA in supercoiled conformation are widely used in low-energy irradiations with LEEs [15, 16], photons [17], and ions [18]. They provide a simple system to evaluate the direct interaction of short-range radiations with DNA, despite the complexity of the molecule. Although purified prokaryotic DNA differs from eukaryotic DNA in terms of supercoiling and the presence of N6-methyladenine [19, 20], supercoiled plasmid DNA offers the advantage of very high sensitivity for the detection of single- and double-strand breaks. One of the main concerns with plasmid DNA films is maintenance of the DNA integrity during film preparation [21]. When the irradiation target is supercoiled DNA, the proportion of the supercoiled configuration is often used as a measure of DNA integrity. The DNA molecule is very sensitive to conditions such as temperature, humidity, and pH, hence, the DNA films must be prepared under well-controlled conditions to minimize damage. The concentration of ions in the solution of DNA has also a considerable influence in maintaining the DNA during film preparation [21, 22]. Furthermore, the type of substrate on which DNA is deposited affects the integrity of the molecule. Among the various substrates tested including tantalum (Ta), gold and graphite, Ta induces the least damage to DNA [23]. 

Pt compounds such as cisplatin and carboplatin bind to the N7 atom of purine bases and produce the Pt-DNA adducts including mainly intrastrand cross-links, interstrand cross-links, and monofunctional binding to guanine [24]. The adducts distort the DNA conformation and reduce the structural stability of DNA [24, 25]. Moreover, DNA must tolerate the incubation conditions required to react with Pt compounds. In most in vitro studies, a DNA solution is mixed with a solution of the Pt compounds at 37°C for 24 or 48 hours [26–30]. These conditions affect the integrity of the DNA as a result of depurination and oxidation processes [31]. To maximize the amount of the Pt compounds bound to DNA while keeping the DNA intact, all parameters involved in the preparation of the films must be known and carefully controlled. In particular, experimental conditions for the reaction of Pt compounds with DNA must be determined as well as the effect of chemical binding of Pt compounds on the stability of DNA. 

In the present study, we investigate the parameters of the Pt compounds and platination reactions on DNA integrity in the preparation of cisplatin/DNA and carboplatin/DNA films. Optimum experimental conditions are determined to retain a high proportion of the supercoiled form of plasmid DNA in Pt-DNA films.

## 2. Experimental Section

### 2.1. Preparation of Plasmid DNA

Plasmid DNA (pGEM-3Zf(-), 3197 base pairs, ca. 1968966 amu per plasmid) was extracted from Escherichia coli JM109 and purified with a HiSpeed plasmid Maxi kit (QIAGEN) [32]. The purified plasmid DNA consisted of 96% supercoiled, 2% cancatemeric, and 2% nicked circular forms. The concentration of DNA and the relative quantity of proteins in the plasmid DNA solution was then calculated by measuring the ratio of ultraviolet (UV) absorption of DNA and protein at 260 nm and 280 nm, respectively, with a Synergy HT-I spectrophotometer. The ratio was 1.98 which corresponds to a purity greater than 85% [33]. The TE buffer (Tris-EDTA: 10 mM–1 mM) was separated from DNA by gel filtration with a Sephadex G-50 medium [34]. Thus the final solution consisted of DNA and ddH_2_O after the filtration. To evaluate the effect of Tris on the binding of Pt compounds to DNA, two different groups of the DNA solutions were prepared. In the first group, Tris buffer was added to the DNA solution at the ratio of the one tris molecule per nucleotide, and in the second group, the DNA solution was prepared with ddH_2_O alone. The DNA concentration was the same in both groups. In each group, control samples were kept in the temperature of −20°C and quantified for the analysis of temperature effect on DNA.

### 2.2.  Platination  of  Plasmid  DNA

The  Pt  compounds, cisplatin [*cis*-diamminedichloroplatinum(II)] and carboplatin [*cis*-diammine(1,1-cyclobutanedicarboxylato)platinum(II)], were purchased from Sigma-Aldrich with a stated purity of 99.9% and ≥98%, respectively, and used without further purification. Their solutions were prepared in ddH_2_O in different concentrations based on their molar solubility. Reactions of cisplatin and carboplatin with the DNA solutions were performed under diverse experimental conditions. These consisted of (1) two different incubation temperatures, that is, 37°C and 25°C, (2) incubation times varying from 40 minutes to 24 hours, and (3) molar ratios between Pt compounds and DNA varying from ratios 2 : 1 up to 200 : 1. DNA platination reactions were performed in the dark to inhibit photoaquation processes as aqueous solutions of cisplatin and carboplatin are degraded via illumination, especially at wavelengths below 500 nm [35, 36]. To terminate the reactions after a given incubation time, the solutions were passed through a gel filtration medium packed into a column. By the filtration, the unbound Pt compounds, tris molecules, and complexes of tris with Pt compounds were separated from the Pt-DNA solutions. The solutions passed through the homemade column packed with Sephadex-G50 gel on a glass bead bed. Sephadex G-50 is a suitable medium for separation of the molecules having a molecular weight larger than 3 × 10^4^ g mol^−1^ from molecules with a molecular weight smaller than 1500 g mol^−1^. Such filtration is expected to produce clean solutions of Pt-DNA in ddH_2_O because the molecular weights of most undesired compounds and complexes found in the solutions during platination have the molecular weight smaller than 1500 g mol^−1^.

### 2.3. Analysis of Platinum-DNA Binding

The concentration of platinum in the solutions was measured by Elan DRC II inductively coupled plasma mass spectroscopy (ICPMS, from Perkin Elmer) which has been used as a suitable method for measurement of platinum in many biomedical applications [37, 38]. Additionally, three control samples consisting of the Pt compounds dissolved in ddH_2_O at known concentrations were also prepared to calibrate the ICPMS measurements of Pt-DNA samples. The DNA concentration was measured by spectrophotometry. It was determined from the optical density of DNA in solution measured by UV absorption at a wavelength of 260 nm. The concentration of DNA was calculated from the reference optical density.

### 2.4. Preparation of Substrate, DNA, and Pt-DNA Films

The DNA and Pt-DNA samples were deposited on a Ta substrate. As shown in previous studies, the stability of supercoiled plasmid DNA on Ta substrate is acceptable for vacuum experiments on LEE-induced damage [23, 39]. The Ta substrates in the current work consist of a thin film of Ta of thickness 450 ± 50 nm evaporated onto a 0.4 mm thick silicon wafer. The surface of Ta was cleaned in pure ethanol and ddH_2_O and dried with a flow of dry nitrogen. Before deposition of DNA and Pt-DNA samples onto the substrate, the TE buffer was added to the DNA and Pt-DNA solutions in the ratio of 3 : 1 (three organic ions per nucleotide). It has been shown that this ratio protects the supercoiled form of DNA during the process of DNA film preparation [22]. The volumes of 7 *μ*L of the latter solutions of DNA and Pt-DNA consisting of 250 ng of each complex (DNA and TE molecules as well as Pt-DNA and TE molecules) were deposited onto the cleaned Ta surface. These quantities were calculated to allow formation of a five-monolayer film (about 10 nm thickness) on the Ta substrate. Such a thickness has been widely used in DNA-LEE experiments because it is smaller than the effective range of these electrons (12–14 nm) for damaging DNA [40]. After freezing at −65°C for 10 minutes in a glove box, the samples were lyophilized (freeze-dried) under a pressure of 7 mTorr by a hydrocarbon-free turbomolecular pump for 2 hours. 

### 2.5. Quantification of the DNA and Pt-DNA Films

The DNA and Pt-DNA films were recovered from the Ta substrates with 10 *μ*L of TE buffer. Comparison of the amount of recovered DNA with the original DNA solution used for deposition showed that approximately 98% of DNA was recovered by the TE buffer. Quantification of the different structural forms (e.g., supercoiled, nicked circular, linear, etc.) in the DNA and Pt-DNA samples was performed by agarose gel electrophoresis. The DNA samples and the agarose gels were stained with SYBR Green I in the concentration of 100x and 10000x, respectively. The samples were run on 1% agarose gel in 1x TAE buffer at 100 volts for 7 minutes following by 75 volts for 68 minutes (5 V cm^−1^). The gels were then scanned by Typhoon-Trio laser scanner (from GE Healthcare) adjusted for the blue fluorescent mode at an excitation wavelength of 488 nm and filter type 520 nm-bandpass (520 BP 40) in the normal sensitivity mode. Various forms of the DNA such as supercoiled, nicked circular, etc. were analyzed by ImageQuant 5.0 (Molecular Dynamics) software. To accurately quantify, the binding efficiencies of SYBR Green I for the same amount (75 ng) of supercoiled and linear DNA were measured, and then the correction factor was determined. This factor arises from the weaker binding of SYBR Green I to supercoiled DNA than to the nicked circular and linear forms. A correction factor of 1.2 was obtained and applied to the quantification of plasmid DNA.

### 2.6. Statistical Analysis

OriginPro 8.1 SR1 (OriginLab Corporation) software was used for statistical and mathematical analysis. Paired *t*-test was the statistical test in which a probability of 0.05 (5%) has been considered significant.

## 3. Results and Discussion

### 3.1. Effects of Incubation Temperature on DNA and Pt-DNA Samples

**Figure 1:**
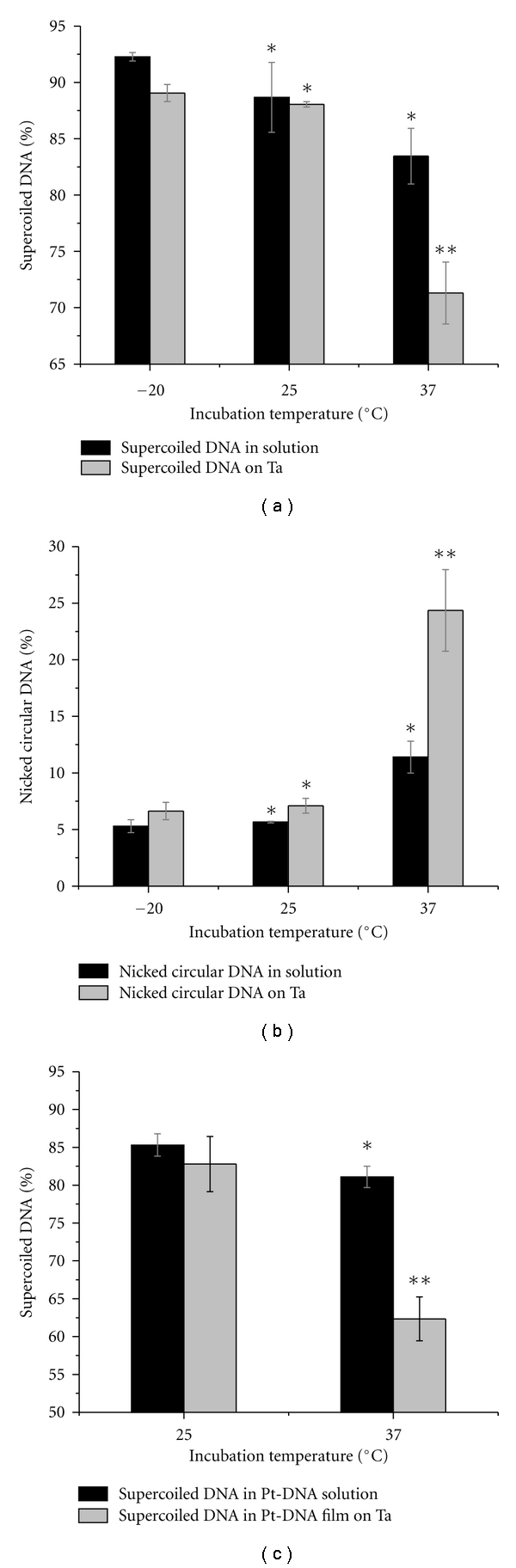
Comparison of the percentages of DNA supercoiled (a), DNA nicked circular (b), and Pt-DNA supercoiled (c) forms in the solution and film samples after incubation at −20°C, 25°C, and 37°C for 24 hours. Data in (a)–(c) are means from three independent experiments; three samples at each temperature are analyzed in each experiment; error bars show standard deviations. *indicates *P* value >0.05, **indicates *P* value <0.05.

Figure 1 (panels a and b) shows a comparison of the percentage of supercoiled and nicked circular forms of the DNA in the samples that had been incubated for 24 hours at three different temperatures: −20°C, 25°C, and 37°C. For each incubation temperature, DNA analysis was performed for two types of samples: (i) “DNA solutions”, that is, samples obtained directly from the incubated solutions, and (ii) “DNA films”, that is, samples that had, after incubation, been deposited and recovered from a Ta substrate. TE buffer was added to the samples at a concentration corresponding to three organic ions per nucleotide. Increasing the incubation temperature resulted in a reduction of the supercoiled form of DNA in both the solution and the film samples. The decrease is relatively small for the DNA solution samples; the samples incubated at 25°C and 37°C show a decrease of 3.8% and 9.5%, respectively, relative to that seen in the sample maintained at −20°C. At each temperature, the DNA samples recovered from Ta show a greater loss of supercoiled DNA than do the samples analyzed directly from solution. A fraction of the supercoiled loss in the film samples is related to the damages which were induced during the incubation in solution. Consequently, for DNA recovered from Ta, a decrease in the supercoiled form with increasing temperature is also observed, and the decrease is very large for the samples incubated at 37°C. The decreases in the supercoiled form are not statistically significant among the DNA solution samples with different incubation temperatures (*P* value: 0.314, 0.106). However, the difference is statistically significant between the DNA film samples incubated at 37°C and the DNA films from samples incubated at 25°C and −20°C (*P* value: 0.012 and 0.009). Additionally, there is no significant difference between the DNA films incubated at 25°C and −20°C (*P* value: 0.136). 

As expected, there are enhancements in the formation of the nicked circular form with increasing incubation temperature. The increase is small except for the DNA film samples which were incubated at 37°C. In these samples the nicked circular form increases by factors of 3.7 and 3.4 compared to those kept at −20°C and 25°C, respectively. These differences are statistically significant (*P* value: 0.02 and 0.011). The high proportion of the nicked circular form in the DNA recovered from films introduces considerable inaccuracy in the evaluation of radiation-induced DNA damage.

In vitro studies have shown that heat can induce various types of DNA damage such as depurination and guanine oxidation mediated by reactive oxygen species (ROS) [31, 41]. Reaction rate constants for formation of 8-oxoguanine and guanine depurination at 37°C are 4.7 × 10^−10^ s^−1^ and 1.3 × 10^−9^ s^−1^ in DNA solutions, respectively [41]. In our experiment, each plasmid sample contained 0.065 pmole of DNA bases in a volume of 7 *μ*L. After a 24-hour incubation of the plasmid DNA at 37°C, we can estimate that approximately 7% and 18% of the plasmid contain 8-oxoguanine molecules or have undergone guanine depurination, respectively. Such DNA molecules are more susceptible to strand breakage than the original DNA. Furthermore, evacuation and lyophilisation during film preparation induce physical stress and can damage DNA [21]. Therefore, the DNA molecules, which have been kept at 37°C for 24 hours or more, do not have sufficient structural stability to tolerate the process of film preparation. Our results suggest that the samples incubated at 37°C are more sensitive and vulnerable to the film preparation and recovery processes than DNA samples incubated at 25°C and −20°C.

Figure 1(c) shows the comparison of the percentage concentration of supercoiled forms in samples of cisplatin-DNA complexes incubated at 25°C and 37°C for 24 hours. Again, the analyses were performed for two groups of samples: (i) Pt-DNA solutions and (ii) Pt-DNA films on a Ta substrate. In the solution and film samples, the proportion of the supercoiled form of Pt-DNA is less than those for DNA alone. The molar ratio of cisplatin to DNA in the solutions was 2 : 1. TE buffer was added to the samples in the concentration of three organic ions per nucleotide. Predictably, in both samples the supercoiled form of DNA decreased when the incubation temperature increased. The decrease is small (4.2%) in the samples of Pt-DNA solution. In contrast, there is a large decrease in the supercoiled form of the Pt-DNA film samples (20.5%). Statistical analysis also showed that the decrease is significantly different for the Pt-DNA films with different incubation temperatures (*P* value: 0.0049). According to our results, the incubation temperature during preparation of the Pt-DNA solution is a substantial factor in determining the composition of Pt-DNA films on Ta substrate for use in irradiation experiments. Moreover, the results suggest that a film composed of cisplatin-DNA complexes with a high proportion of intact DNA molecules (supercoiled form) on a Ta substrate can be obtained when DNA platination occurs at 25°C. 

### 3.2. Kinetics of Binding Pt Compounds to DNA

**Figure 2:**
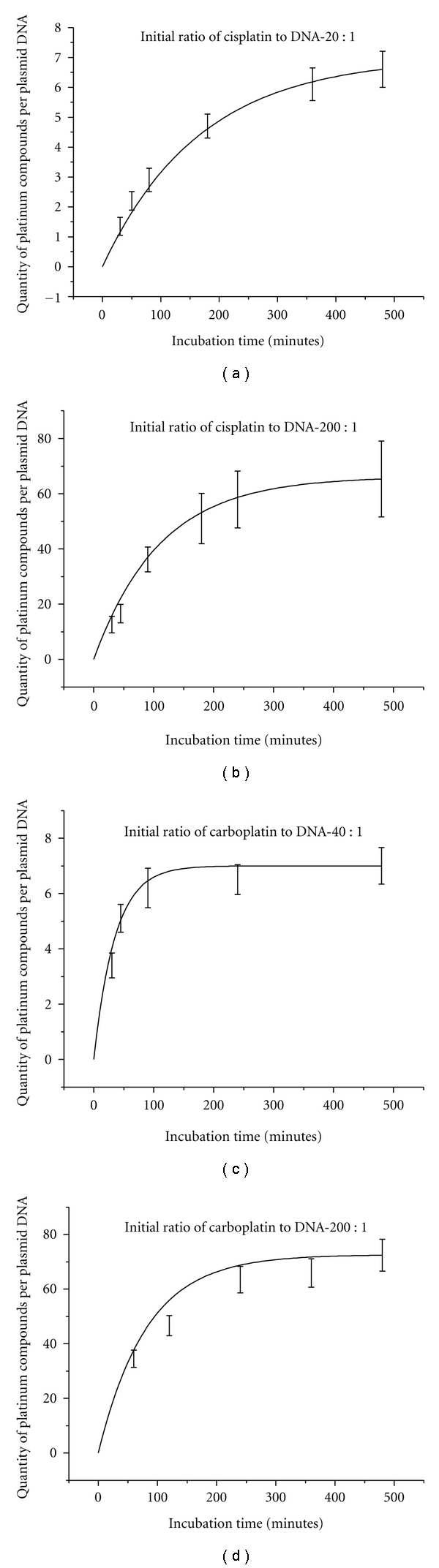
Kinetics of binding of Pt compounds to plasmid DNA. The Pt compounds are: (a) cisplatin with the initial ratios in the solution of 20 : 1, (b) 200 : 1, and (c) carboplatin with the initial ratios of 40 : 1 and (d) 200 : 1. The curves show the quantity of bound Pt compounds per DNA molecule at different incubation times at 25°C. Data in (a)–(d) are means from three measurements; error bars show standard deviations. The continuous black lines are exponential fits to the data.

Following platination at 25°C, DNA has much less damage during the process of deposition and recovery from the Ta substrate. However, the DNA platination reaction proceeds with a slower rate. Increasing the concentration of the Pt compounds can compensate for this lower rate.  Figure 2 shows the ratios of bound Pt-compound to DNA for different incubation times at 25°C when the initial concentration ratios of Pt compounds to DNA in solution are 200 : 1, 40 : 1, and 20 : 1. The solution consists of plasmid DNA, cisplatin or carboplatin, and tris with the ratio of 1 : 1 nucleotide. This amount of tris was considered as the minimum amount of buffer which can preserve the stability of DNA during the preparation process. It is clearly seen that the binding kinetics of cisplatin and carboplatin to DNA are similar and exhibit exponential behavior. These curves generally reach saturation prior to 8 hours and show a linear behaviour prior to 2 hours. For the initial concentration ratio of 200 cisplatin molecules per DNA, it is possible to have Pt-DNA samples with the ratios of bound cisplatin to DNA from 16 : 1 to 37 : 1 in 40-minute to 120-minute incubation times, respectively. For the same incubation times, the ratios are 2 : 1 and 3 : 1 when the initial ratio of cisplatin to DNA decreases an order of magnitude (20 : 1). The results demonstrate that various ratios of bound cisplatin or carboplatin to DNA can be obtained in the incubation times of less than 2 hours by increasing the initial concentration of the Pt compounds. Since the kinetics curves obey a linear fit for these incubation times, it is possible to simply extrapolate a variety of Pt-DNA ratios from this part of the curves.

**Figure 3:**
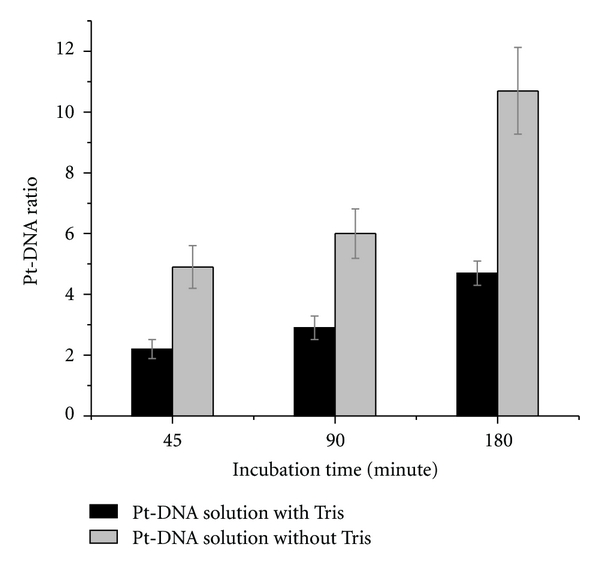
Impact of tris on the reaction of DNA platination. Pt-DNA ratios in the cisplatin-DNA solutions incubated during 45, 90, and 180 minutes at 25°C are compared in the presence and absence of tris. Data are means from three measurements; error bars show standard deviations.

Since Pt compounds can react with most buffers [42], their concentration is also a relevant parameter in the DNA platination process (i.e., buffers compete with DNA for binding Pt compounds). Tris is widely used as a buffer, especially for solutions of nucleic acids. It also reacts with Pt compounds to produce *cis*-[Pt(NH_3_)_2_(N-Tris)(OH)]^+^ and *cis*-[Pt(NH_3_)_2_(N,O-TrisH_−1_)]^+^ [43]. The bar graphs in Figure 3 show a comparison of bound Pt compounds to DNA ratios for three different incubation times at 25°C for two different solutions: (i) a mixture of DNA, cisplatin, and ddH_2_O, and (ii) a mixture of DNA, cisplatin, ddH_2_O, and tris with the concentration ratio of 1 : 1 nucleotide. The initial concentration ratio of cisplatin to the DNA was 20 : 1 in the solutions. The results demonstrate that the ratio of bound cisplatin to the DNA is more than double when the platination reaction occurs in a ddH_2_O solution without tris molecules.

### 3.3. Effects of Incubation Time on DNA and Pt-DNA Films

**Figure 4:**
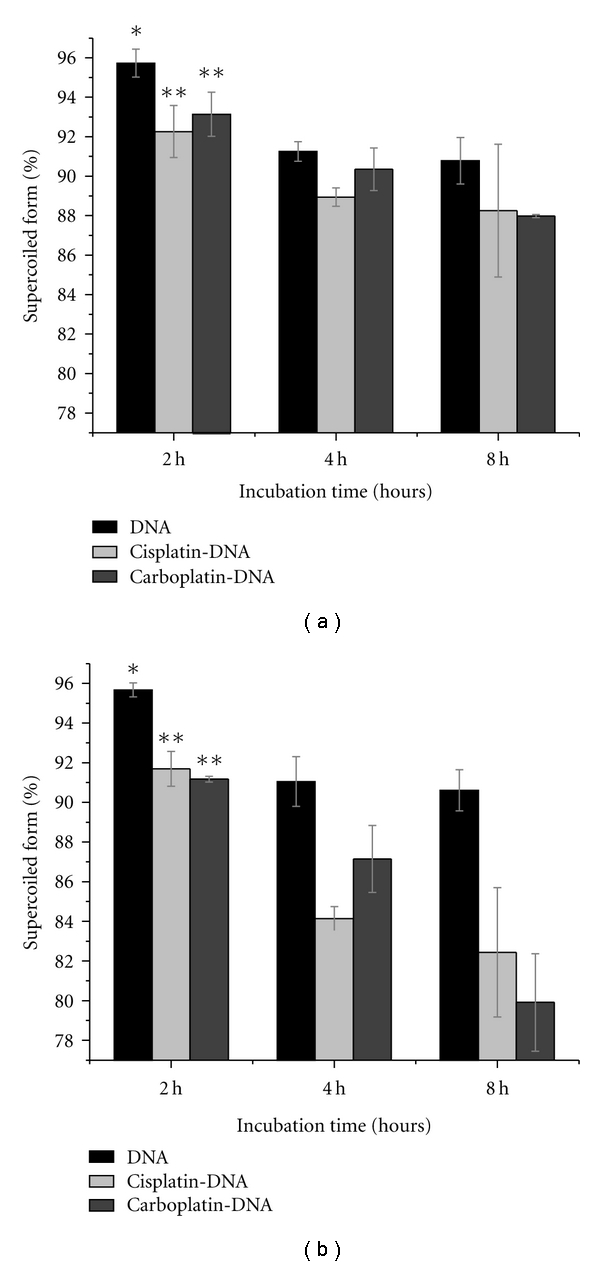
Comparison of the percentages of supercoiled forms in the samples of DNA, cisplatin-DNA, and carboplatin-DNA (a) in solution, and (b) on Ta substrate, after incubation for 2, 4, and 8 hours at 25°C. Data are means from three measurements; error bars show standard deviations.*indicates *P* value >0.05, **indicates *P* value <0.05.

The bar graphs in Figure 4 show a comparison of the percentage of supercoiled DNA and Pt-DNA samples that were incubated at 25°C for 2, 4, and 8 hours. The analyses were performed for samples that had been recovered (i) from solution, immediately after incubation (Figure 4(a)), and (ii) from films deposited on Ta (Figure 4(b)). The Pt-DNA samples were prepared with either cisplatin or carboplatin. The initial concentration ratio of the Pt compounds to DNA was 200 : 1 and that of the TE buffer was three organic ions per nucleotide. As seen from Figure 4, more than 90 percent of the DNA, in samples incubated for 2 hours, is in the supercoiled form. The proportion of supercoiled form decreases when the samples are incubated for 4 hours or more. The decrease is statistically significant in all samples except for the pure DNA solution sample. As might be expected, the decrease is greater in Pt-DNA films than in DNA samples. Thus, it is possible to prepare Pt-DNA films with a high proportion of supercoiled DNA at various ratios of bound Pt to DNA, by mixing DNA with high concentrations of Pt-compound solution and restricting the length of the incubation to less than 2 hours, as long as the incubation temperature does not exceed 25°C.

### 3.4. Effects of Bound Pt to DNA on Pt-DNA Samples Analysis

**Figure 5:**
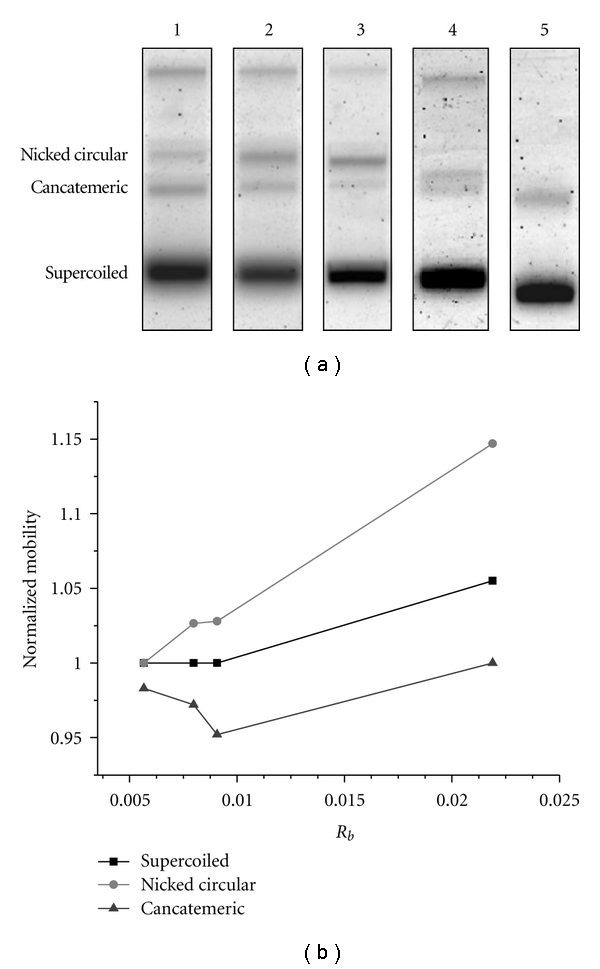
Mobility of cisplatin-DNA molecules in agarose gel. (a) Migration of the different configurations of cisplatin-DNA molecules separated by electrophoresis. Lane 1 is for a DNA sample and lanes 2–5 are for cisplatin-DNA samples with the number of bound cisplatin molecules per nucleotide, *R*_*b*_, of 0.0057, 0.008, 0.0091, and 0.0219, respectively. (b) Normalized mobility of the nicked circular, supercoiled, and cancatemeric forms of Pt-DNA samples at different *R*_*b*_ in gel electrophoresis.

The distortion of the DNA structure resulting from the formation of Pt-DNA cross-links must be considered in quantification methods such as electrophoresis.  Figure 5(a) shows the migration of different forms of cisplatin-DNA in the electrophoresis gel. The mobility of the nicked circular, cancatemeric, and supercoiled bands is changed with increasing numbers of bound Pt molecules per nucleotide (*R*_*b*_). The change is due to distortion of the different forms of DNA by cisplatin since Pt-DNA crosslinks are known to cause conformational changes in DNA including shortening (bending) and unwinding [44, 45]. The distortion becomes greater as a function of the quantity of bound Pt molecules.  Figure 5 shows the dependence of the mobility of the supercoiled, nicked circular, and cancatemeric forms of cisplatin-DNA samples as a function of the ratio *R*_*b*_ in a 1% agarose gel. The mobility of each form of Pt-DNA is normalized to the same form of an unmodified DNA sample (Figure 5(b)). As seen from Figure 5(b), the migration of the nicked circular and supercoiled configurations generally increases with rising *R*_*b*_. However, the mobility of the nicked circular form increases with a faster rate than that of the supercoiled form. Mobility of the cancatemeric configuration decreases with rising in *R*_*b*_ up to 0.009 and then increases for higher *R*_*b*_. 

Since the number of Pt molecules per plasmid probably represents a Poisson distribution for each Pt-DNA ratio, this would be expected to reduce the resolution of the agarose gels by increasing the dispersion within each band (i.e., the band width). The linear plasmid band lies between the nicked circular and cancatemeric bands; thus an increase in band width could hinder precise quantification of the linear band which usually is weaker than the others. Furthermore, the nicked circular and cancatemeric bands merge owing to increased band width and form one band at *R*_*b*_ = 0.022. Our results show that the mobility changes are substantial for *R*_*b*_ greater than 0.005.

## 4. Conclusion

Thin films of platinum-DNA adducts can be considered as useful models in irradiation experiments to study the molecular mechanisms of radiosensitization which underlie concomitant chemoradiation therapy. We have investigated the optimum experimental conditions to prepare dry thin films of Pt compounds bound to plasmid DNA on a Ta substrate. Incubation conditions in DNA platination reactions have substantial effects on the stability of Pt-DNA, particularly in the thin film samples preparation. In most in vitro experiments, reaction of Pt compounds with DNA solutions has been performed at 37°C for incubation times varying from 24 to 48 hours. However, our results show that these conditions can induce damage to the DNA and highly sensitize them to manipulations required to form thin films and recover DNA from the Ta substrate. The concentration of intact DNA increases significantly in the film samples when the incubation temperature during reaction with the Pt is reduced to 25°C and the time of incubation is 2 hours. By increasing the concentration of the Pt compounds, it is possible to compensate for the reduced reaction rate at lower temperature. High levels of plasmid platination however affect the quantification of Pt-DNA samples in agarose gel electrophoresis, because Pt-DNA adducts distort the conformation of DNA molecules. Therefore, the optimum condition is obtained from an equilibrium between temperature, time, and Pt compounds concentration during the DNA platination reaction. 

By recording the kinetics of binding Pt compounds to DNA, it is possible to extrapolate different Pt-DNA ratios from the kinetics curves. We have found that the proportion of supercoiled DNA is more than 90% in the Pt-DNA film when the DNA platination reaction is performed at 25°C for less than 2 hours in solutions containing the Pt compound with quantities of less than 3 × 10^−2^ Pt molecules per nucleotide and the minimum concentration of Tris buffer (one tris molecule per nucleotide). Under these conditions, agarose gel electrophoresis is an accurate method for quantification of DNA damage. We have also determined that the maximum number of bound Pt-compound per nucleotide is about 5 × 10^−3^ under our optimum conditions. This ratio is an order of magnitude higher than those found in biological studies and clinical applications [46]. These high ratios, however, are useful for in vitro mechanistic studies in which substantial quantities of product are required. Hence, we have found that by adjusting the initial concentration of Pt compounds in solution, Pt-DNA films having a known controlled ratio of platinum chemotherapeutic agents to DNA can be obtained while maintaining DNA integrity.
